# Supplementary material for: Effect of a Remotely Delivered Weight Loss Intervention in Early-Stage Breast Cancer: Randomized Controlled Trial
Source: Nutrients. 2021 Nov 15;13(11):4091. doi: 10.3390/nu13114091 (PMC8622393; doi:10.3390/nu13114091)
Supplement: Supplementary file 1 [file nutrients-13-04091-s001.zip › nutrients-1448501-supplementary.pdf]

SUPPLEMENTARY MATERIAL

Table S1. Criteria for the clinical diagnosis of metabolic syndrome in women and calculation of continuous metabolic syndrome risk score in Living Well after Breast Cancer

	Metabolic Syndrome (harmonized definition)[1]	Continuous Metabolic Syndrome risk score[2,3]				
		Sample[a]	Population-based[b]		Mean for z score[c]	SD for z score[d]
		Mean	Mean (adults)	Mean (age matched women)		
Components	***Threshold for met component***					
Waist Circumference, cm	≥80 cm	105.81	87.6	88.91	1.95	0.04
Triglycerides, mmol/L	≥1.7 mmol/L or specific treatment for this lipid abnormality[e]	1.47	1.3	1.20	0.08	0.22
HDL cholesterol, mmol/L	<1.3 mmol/L or specific treatment for this lipid abnormality[e]	1.44	1.4	1.68	0.22	0.10
Systolic and Diastolic blood pressure, mmHg[f]	Systolic ≥130 and/or diastolic ≥85 mmHg or treatment of previously diagnosed hypertension	101.32	97.7	97.03	1.99	0.04
Fasting glucose, mmol/L	≥5.6 mmol/L or previously diagnosed type 2 diabetes	5.59	5.4	5.31	0.72	0.07
**Summary scores**	***Metabolic Syndrome***	***Continuous Metabolic Syndrome risk score***				
Present	≥3 components met	= ((log10(waist circumference) – 1.95) / 0.04 + (log10(triglycerides) – 0.08) / 0.22 + (0.22 – log10(HDL)) / 0.10 + (log10(mean of systolic & diastolic blood pressure) – 1.99) / 0.04 + (log10(glucose) – 0.72) / 0.07) / 5				
Absent	<3 components met & 5 measured					
Unknown	All else					

Abbreviations: HDL, high-density lipoprotein; mmHg, millimetres of mercury; mmol/L, millimoles per litre.
[a] Baseline mean in the Living Well after Breast Cancer sample n=159
[b] From Australian population-based study (2011-2012 Australian Diabetes, Obesity and Lifestyle study) of 4,614 adult attendees, displaying reported means for adults[4] and mean in age-matched women, obtained by author request[4] with data available for n=2,546 women (triglycerides, HDL cholesterol, blood pressure), n=2,544 (waist circumference) and n=2541 (fasting glucose). The 5-year age-bands and weights used were: 4/159 (<40 y); 10/159 (40–<45 y); 29/159 (45–<50 y); 44/159 (50–<55 y); 21/159 (55–<60 y); 19/159 (60–<65 y); 19/159 (65–<70 y); 13/159 (70–<75 y); and 0/159 (≥75 y).
[c] Mean in age-matched women of log10 transformed biomarker
[d] SD in sample of log10 transformed biomarker
[e] If HDL-cholesterol and/or triglycerides were inferred from self-report lipid lowering medication-use (yes/no), only one component was counted, as it could not be reasonably certain that both dyslipidemic conditions were actually present.
f Mean of systolic and diastolic blood pressure

Table S2. Variables included in multiple imputation models (outcomes imputed separately by chained equations, STATA 16, m=50 imputations)

Variable	Associated with dropout / missingness at $p<0.05$	Weight	Waist circumference	Fat Mass	Lean Mass	MS Risk score	Triglycerides	HDL	Systolic BP	Diastolic BP	Glucose	Phys QOL	Mental QOL	Fatigue	GCS - Psychological	GCS - Somatic	GCS - vasomotor	Fear of Recurrence	Body Image	Musculoskeletal Pain
Age (y)	D, B, LA									A	A				A			A	A	
Physical QOL (T Score)	D, DX, B, LA		A	A																A
Weekly gross household income (AUD: <\$82,056 / ≥\$82,056 / unknown)	D, B, LA	A	A	A	A	A								A	A			A	A	A
Treatment (surgery only / surgery + chemotherapy / surgery + radiotherapy / surgery + radiotherapy + chemotherapy)	D, B, LA		A		A		A	A					A	A	A		A			
Depression and/or anxiety (yes / no)	D, DX, LA					A	A	A	A	A	A	A		A		A				A
Children <18 at home (yes / no)	D, DX, B												A	A				A		
Mastectomy (yes / no)	DX, LA	A		A		A						A			A			A		
Endocrine Treatment (none / aromatase inhibitor / other) [a]	DX, B, LA		A		A	A	A	A	A	A	A	A		A		A		A		
Lymphoedema (yes / no)	DX, LA	A	A	A		A							A		A			A	A	
Diabetes (yes / no)	DX, LA												A		A				A	

Table S2. Variables included in multiple imputation models (outcomes imputed separately by chained equations, STATA 16, m=50 imputations) (continued)

Variable	Associated with dropout / missingness at p<0.05	Weight	Waist	Fat Mass	Lean Mass	MS Risk	Triglycerides	HDL	Systolic BP	Diastolic BP	Glucose	Phys QOL	Mental QOL	Fatigue	GCS - [illegible]	GCS - [illegible]	GCS - [illegible]	Fear of [illegible]	Body Image	Musculoskele[illegible]
Positive Lymph Nodes (yes / no)	Q, LA	A		A		A						A				A				A
Ethnicity (Caucasian / other)	LA		A	A						A	A	A								A
Menopausal Status (pre- / peri- / post- menopausal)	LA	A		A	A	A			A	A	A		A	A		A	A	A	A	
Breast Cancer Stage (1 / 2 / 3)	LA		A			A									A		A	A	A	A
Married or stable union (yes / no)	LA			A	A	A			A	A	A				A	A				
Prior weight loss aids (yes / no)	-												A							
Employment (working / retired / other not working)	-				A									A	A				A	A
Body Mass Index (kg/m²)	-				A	A			A	A	A	A		A		A			A	
Depression (T score)	-	A			A								A		A	A	A			
Breast reconstruction (yes / no / planned)	-			A		A	A	A	A						A			A	A	A
Time Since Diagnosis (months)	-	A			A		A	A												
Education (≤ high school / technical or diploma / ≥ university)	-	A			A				A							A	A			
Country of Birth (Australia / other)	-	A	A	A	A	A						A	A			A			A	
Charlson Comorbidity Index (0 / 1 / 2 / 3 / ≥4)	-		A	A									A				A	A		A

Table S2. Variables included in multiple imputation models (outcomes imputed separately by chained equations, STATA 16, m=50 imputations) (continued)

Variable	Associated with dropout / missingness at p<0.05	Weight	Waist	Fat Mass	Lean Mass	MS Risk	Triglycerides	HDL	Systolic BP	Diastolic BP	Glucose	Phys QOL	Mental QOL	Fatigue	GCS -	GCS -	GCS -	Fear of	Body Image	Musculoskele
Smoking status (every smoked: yes / no)	-	A		A	A	A	A	A				A	A					A		
Moderate-Vigorous Physical activity (min/week)	-					A	A	A				A								
Fatigue (score on FACIT scale)	-	A			A	A	A	A				A			A		A	A	A	A
Breast cancer subtype (HR+ and HER2- / HR+ and HER2- / HR+ and HER2+ / HR- and HER2+ / unknown) [b]	-	A			A		A	A	A			A	A	A	A					
Blood pressure tablets (yes / no)	-								A					A						
Cholesterol tablets (yes / no)	-		A						A			A			A					A

Abbreviations: AUD, Australian dollar; BP, blood pressure; FACIT, Functional Assessment of Chronic Illness Therapy; HDL, High Density Lipoprotein; HER2, Human epidermal growth receptor 2; HR, hormone receptor; GCS, Greene Climacteric Scale; MS, metabolic syndrome; QOL, quality of life

Grey = included in imputation model as a predictor of dropout (D; eTable 4) or additional sources of missingness concerning: unavailability of the DXA machine (DX); not completing the fasting blood / blood pressure assessment (B); skipping the Questionnaire or Questionnaire items (Q); and, the late addition of the measure to the trial assessment battery (LA). Non-English Speaking Background (not shown) was excluded from imputation models regardless of association with dropout / missing data due to low numbers.

A = included in imputation model as auxiliary variable to improve prediction of the missing outcome variables (associated with the outcome at p<0.2 in backwards elimination).

[a] Other is all selective estrogen receptor modulators except for one gonadotropin releasing hormone agonist

[b] Included using the smallest categories collapsed due to insufficient numbers (HR- and HER2+ combined with unknown)

Table S3. Predictors of drop-out [a]

Variable	Completer (n=124)	Drop-out (n=35)	p
	Mean ± SD or n (%)		
**Access: Time availability**			
Age (years) [b]	56.3 ± 8.8	52.4 ± 10.0	**0.03**
Children <18 years at home	21 (16.9%)	12 (34.3%)	**0.03**
Employment			0.73
Working	73 (58.9%)	21 (60.0%)	
Retired	28 (22.6%)	6 (17.1%)	
Other not working	23 (18.6%)	8 (22.9%)	
Married or stable union	89 (71.8%)	21 (60.0%)	0.19
**Access: Socioeconomic Status**			
Gross Household Income (AUD) [c]			**0.007**
<$82,056 per year	51 (41.1%)	20 (57.1%)	
≥$82,056 per year	56 (45.2%)	15 (42.9%)	
Not reported/not known/missing	17 (13.7%)	0 (0%)	
Non-English speaking background	4 (3.2%)	5 (14.3%)	**0.02**
Caucasian	122 (98.4%)	34 (97.1%)	0.64
Born in Australia	98 (79%)	25 (71.4%)	0.34
Education			0.36
High school or less	50 (40.3%)	14 (40.0%)	
Technical / Trade / Diploma	26 (21.0%)	11 (31.4%)	
University or higher	48 (38.7%)	10 (28.6%)	
**General health**			
QOL Physical Health Component (T score) [b]	46.0 ± 6.4	43.0 ± 7.4	**0.02**
Depression (T score)	50.2 ± 8.6	51.2 ± 8.7	0.54
BMI (kg/m^2)	31.3 ± 5.2	31.6 ± 4.6	0.77
Smoking status [c]			0.86
Never smoked ≥100 cigarettes in lifetime	72 (58.1%)	20 (57.1%)	
Previously smoked	46 (37.1%)	14 (40.0%)	
Currently smoker	6 (4.8%)	1 (2.9%)	
Metabolic Syndrome present	59 (48.0%)	15 (46.9%)	0.91
Blood pressure medications	30 (24.2%)	5 (14.3%)	0.22
Cholesterol medication	19 (15.3%)	5 (14.3%)	0.88
**Breast Cancer Characteristics**			
Months since diagnosis	10.8 ± 5.1	10.4 ± 4.9	0.71
Months since treatment completion	5.3 ± 4.9	4.1 ± 3.3	0.19
Menopausal status at diagnosis			0.41
Premenopausal	43 (34.7%)	16 (45.7%)	
Perimenopausal	16 (12.9%)	5 (14.3%)	
Postmenopausal	65 (52.4%)	14 (40.0%)	
Breast cancer stage [d]			0.29
Stage 1	71 (57.3%)	15 (44.1%)	
Stage 2	41 (33.1%)	13 (38.2%)	
Stage 3	12 (9.7%)	6 (17.7%)	

Table S3. Predictors of drop-out [a] (continued)

Variable	Completer (n=124)	Drop-out (n=35)	p
	Mean ± SD or n (%)		
ER positive [d]	109 (87.9%)	30 (88.2%)	0.96
HER2 positive [d]	13 (10.5%)	7 (20.6%)	0.12
Treatment [b, c]			**0.04**
Surgery only	7 (5.7%)	3 (8.6%)	
Surgery and Chemotherapy	22 (17.7%)	1 (2.9%)	
Surgery and Radiation	41 (33.1%)	9 (25.7%)	
Surgery, Chemotherapy and Radiation	54 (43.6%)	22 (62.9%)	
Endocrine treatment [c]			0.11
None	48 (38.7%)	19 (54.3%)	
SERM	31 (25.0%)	10 (28.6%)	
Aromatase inhibitors	44 (35.5%)	6 (17.1%)	
GnRH agonist	1 (0.8%)	0 (0.0%)	

Abbreviations: AUD, Australian dollar; BMI, body mass index; ER, estrogen receptor; GnRH, gonadotropin releasing hormone; HER2, Human epidermal growth receptor 2; SERM, selective estrogen receptor modulator; QOL, quality of life.

[a] Participants were classed as completer if they participated in follow-up assessment of the primary outcome at all follow-up assessments, otherwise as a drop-out.

[b] Selected indicator for inclusion in multiple imputation models ($p<0.05$ and best indicator within category)

[c] Alternative test used due to small numbers, likelihood ratio chi-square; collapsed categories: endocrine treatment (none / aromatase inhibitors / SERM or GnRH agonist); smoking status (never smoked / previously or currently smoke)

[d] Percentages exclude 1 participant (drop-out) with missing pathological data.

Table S4. Changes in primary and secondary outcomes in the Living well after Breast Cancer Trial (multiple imputation analysis)

Outcome	Timepoint	Intervention (n=80)	Usual care (n=79)	Intervention effect (Intervention - Usual care)		
		**Mean change (95% CI)** [a]	**Mean change (95% CI)** [a]	**Difference (95% CI)** [a]	**p**	**D**[b]
Weight (% of baseline value)	Baseline M (SD)	83.92 (14.19)	83.64 (13.62)			
	6-months	-4.59 (-5.82, -3.37)	-0.27 (-1.54, 1.00)	-4.32 (-6.10, -2.54)	**<0.001**	-0.31
	12-months [c]	-5.01 (-6.51, -3.51)	-0.30 (-1.82, 1.21)	-4.71 (-6.84, -2.58)	**0.000**	-0.34
	18-months	-3.66 (-5.26, -2.05)	-0.65 (-2.32, 1.02)	-3.01 (-5.29, -0.73)	**0.010**	-0.22
Weight (kg)						
	Baseline M (SD)	83.92 (14.19)	83.64 (13.62)			
	6-months	-3.75 (-4.78, -2.73)	-0.24 (-1.30, 0.82)	-3.51 (-5.00, -2.02)	**0.000**	-0.25
	12-months [c]	-4.12 (-5.36, -2.88)	-0.31 (-1.57, 0.95)	-3.81 (-5.57, -2.04)	**0.000**	-0.27
	18-months	-3.02 (-4.38, -1.65)	-0.59 (-2.02, 0.84)	-2.43 (-4.37, -0.48)	**0.014**	-0.18
Total fat mass (kg)						
	Baseline M (SD)	39.00 (10.56)	37.36 (10.72)			
	6-months	-2.90 (-3.81, -2.00)	0.06 (-0.90, 1.02)	-2.96 (-4.29, -1.63)	**0.000**	-0.28
	12-months [c]	-2.99 (-4.06, -1.92)	0.27 (-0.91, 1.45)	-3.26 (-4.88, -1.65)	**0.000**	-0.31
	18-months	-1.74 (-2.98, -0.49)	-0.09 (-1.45, 1.27)	-1.65 (-3.43, 0.13)	0.070	-0.16
Total lean mass (kg)[d]						
	Baseline M (SD)	42.82 (5.14)	43.70 (5.27)			
	6-months	-1.00 (-1.38, -0.63)	-0.22 (-0.62, 0.18)	-0.78 (-1.31, -0.26)	**0.004**	-0.15
	12-months [c]	-1.06 (-1.49, -0.63)	-0.42 (-0.92, 0.08)	-0.64 (-1.28, -0.00)	**0.049**	-0.12
	18-months	-1.13 (-1.63, -0.64)	0.03 (-0.53, 0.58)	-1.16 (-1.87, -0.44)	**0.002**	-0.22
Metabolic Syndrome risk score	Baseline M (SD)	0.65 (0.60)	0.64 (0.60)			
	6-months	-0.19 (-0.28, -0.10)	0.05 (-0.04, 0.13)	-0.24 (-0.36, -0.12)	**0.000**	-0.40
	12-months [c]	-0.18 (-0.27, -0.08)	0.02 (-0.08, 0.13)	-0.20 (-0.34, -0.06)	**0.006**	-0.33
	18-months	-0.16 (-0.25, -0.06)	0.03 (-0.08, 0.13)	-0.18 (-0.32, -0.05)	**0.009**	-0.31

Table S4. Changes in primary and secondary outcomes in the Living well after Breast Cancer Trial (multiple imputation analysis) (continued)

**Outcome**	**Timepoint**	**Intervention (n=80)**	**Usual care (n=79)**	**Intervention effect (Intervention - Usual care)**		
		**Mean change (95% CI)** [a]	**Mean change (95% CI)** [a]	**Difference (95% CI)** [a]	**p**	**D**[b]
Waist circumference (cm)	Baseline M (SD)	106.72 (11.70)	104.91 (10.37)			
	6-months	-3.27 (-4.83, -1.72)	-0.52 (-2.07, 1.04)	-2.76 (-4.94, -0.57)	**0.013**	-0.25
	12-months [c]	-5.56 (-7.23, -3.89)	-2.03 (-3.75, -0.31)	-3.53 (-5.91, -1.15)	**0.004**	-0.32
	18-months	-5.36 (-6.93, -3.79)	-2.28 (-3.90, -0.65)	-3.09 (-5.34, -0.83)	**0.007**	-0.28
Triglycerides (mmol/L) [e]	Baseline M (SD)	1.42 (0.71)	1.52 (0.88)			
	6-months	-0.38 (-0.46, -0.29)	-0.27 (-0.37, -0.18)	-0.10 (-0.23, 0.03)	0.120	-0.13
	12-months [c]	-0.42 (-0.52, -0.31)	-0.32 (-0.46, -0.19)	-0.09 (-0.26, 0.07)	0.275	-0.11
	18-months	-0.46 (-0.55, -0.37)	-0.36 (-0.47, -0.26)	-0.10 (-0.24, 0.04)	0.175	-0.12
HDL-cholesterol (mmol/L) [d]	Baseline M (SD)	1.45 (0.30)	1.42 (0.35)			
	6-months	0.02 (-0.02, 0.07)	-0.02 (-0.07, 0.03)	0.04 (-0.02, 0.11)	0.162	0.14
	12-months [c]	0.03 (-0.02, 0.08)	-0.02 (-0.08, 0.03)	0.06 (-0.02, 0.13)	0.137	0.18
	18-months	0.05 (0.00, 0.10)	-0.00 (-0.06, 0.05)	0.06 (-0.02, 0.13)	0.125	0.17
Systolic blood pressure (mmHg)	Baseline M (SD)	125.27 (12.18)	123.46 (11.35)			
	6-months	-2.29 (-4.88, 0.30)	3.84 (1.15, 6.53)	-6.13 (-9.91, -2.35)	**0.001**	-0.52
	12-months [c]	1.05 (-1.89, 3.99)	1.48 (-1.62, 4.58)	-0.43 (-4.74, 3.88)	0.845	-0.04
	18-months	2.69 (-0.93, 6.32)	5.67 (1.66, 9.68)	-2.98 (-8.46, 2.50)	0.286	-0.25
Diastolic blood pressure (mmHg)	Baseline M (SD)	78.66 (9.38)	77.95 (7.32)			
	6-months	-0.30 (-2.00, 1.39)	2.64 (0.87, 4.41)	-2.94 (-5.34, -0.54)	**0.017**	-0.35
	12-months [c]	0.75 (-0.97, 2.47)	1.11 (-0.80, 3.02)	-0.36 (-2.94, 2.23)	0.786	-0.04
	18-months	1.23 (-0.68, 3.14)	3.59 (1.58, 5.60)	-2.36 (-5.16, 0.44)	0.099	-0.28

Table S4. Changes in primary and secondary outcomes in the Living well after Breast Cancer Trial (multiple imputation analysis) (continued)

Outcome	Timepoint	Intervention (n=80)	Usual care (n=79)	Intervention effect (Intervention - Usual care)		
		Mean change (95% CI) [a]	Mean change (95% CI) [a]	Difference (95% CI) [a]	p	D[b]
Fasting plasma glucose (mmol/L)	Baseline M (SD)	5.54 (1.24)	5.63 (1.14)			
	6-months	-0.32 (-0.48, -0.17)	-0.21 (-0.37, -0.04)	-0.12 (-0.33, 0.10)	0.300	-0.10
	12-months [c]	-0.16 (-0.31, -0.01)	0.06 (-0.10, 0.22)	-0.22 (-0.44, -0.00)	**0.050**	-0.19
	18-months	-0.12 (-0.29, 0.05)	-0.09 (-0.29, 0.12)	-0.03 (-0.29, 0.24)	0.829	-0.02
QOL Physical Health component (T score) [d]	Baseline M (SD)	44.74 (6.87)	45.90 (6.61)			
	6-months	2.60 (1.39, 3.81)	0.83 (-0.45, 2.12)	1.77 (-0.03, 3.57)	0.054	0.26
	12-months [c]	3.85 (2.48, 5.23)	0.81 (-0.70, 2.32)	3.04 (0.97, 5.12)	**0.004**	0.45
	18-months	1.99 (0.47, 3.52)	0.83 (-0.84, 2.49)	1.17 (-1.07, 3.40)	0.306	0.17
QOL Mental Health component (T score) [d]	Baseline M (SD)	46.07 (7.26)	45.65 (6.42)			
	6-months	1.51 (0.22, 2.80)	0.17 (-1.16, 1.50)	1.35 (-0.54, 3.23)	0.163	0.20
	12-months [c]	2.29 (0.90, 3.67)	0.70 (-0.81, 2.22)	1.58 (-0.42, 3.59)	0.121	0.23
	18-months	0.06 (-1.77, 1.88)	0.55 (-1.33, 2.42)	-0.49 (-3.15, 2.17)	0.716	-0.07
Fatigue [d]						
	Baseline M (SD)	35.35 (9.79)	37.72 (9.51)			
	6-months	3.38 (1.61, 5.14)	0.51 (-1.26, 2.28)	2.86 (0.40, 5.32)	**0.023**	0.30
	12-months [c]	4.73 (2.90, 6.56)	2.12 (0.21, 4.02)	2.62 (-0.01, 5.24)	0.051	0.27
	18-months	2.84 (0.87, 4.80)	1.99 (0.06, 3.92)	0.85 (-1.89, 3.58)	0.544	0.09
Musculoskeletal Pain						
	Baseline M (SD)	1.63 (1.13)	1.69 (1.18)			
	6-months	-0.23 (-0.51, 0.05)	0.32 (0.05, 0.60)	-0.55 (-0.93, -0.18)	**0.004**	-0.48
	12-months [c]	-0.32 (-0.62, -0.03)	0.22 (-0.10, 0.55)	-0.55 (-0.97, -0.13)	**0.011**	-0.48
	18-months	-0.14 (-0.48, 0.21)	0.20 (-0.17, 0.57)	-0.34 (-0.78, 0.10)	0.133	-0.29

Table S4. Changes in primary and secondary outcomes in the Living well after Breast Cancer Trial (multiple imputation analysis) (continued)

**Outcome**	**Timepoint**	**Intervention (n=80)**	**Usual care (n=79)**	**Intervention effect (Intervention - Usual care)**		
		**Mean change (95% CI)** [a]	**Mean change (95% CI)** [a]	**Difference (95% CI)** [a]	**p**	**D**[b]
Menopausal Symptoms – Psychological subscale	Baseline M (SD)	9.97 (6.18)	9.62 (5.71)			
	6-months	-1.33 (-2.58, -0.08)	-0.32 (-1.55, 0.90)	-1.01 (-2.81, 0.79)	0.272	-0.17
	12-months [c]	-2.11 (-3.55, -0.66)	-0.71 (-2.20, 0.78)	-1.39 (-3.53, 0.75)	0.201	-0.24
	18-months	-1.47 (-2.81, -0.13)	-0.27 (-1.69, 1.15)	-1.21 (-3.14, 0.72)	0.220	-0.20
Menopausal Symptoms – Somatic subscale	Baseline M (SD)	5.46 (4.35)	5.13 (4.05)			
	6-months	-0.68 (-1.37, 0.00)	0.45 (-0.25, 1.14)	-1.13 (-2.09, -0.16)	**0.022**	-0.27
	12-months [c]	-0.83 (-1.61, -0.04)	0.17 (-0.61, 0.96)	-1.00 (-2.12, 0.12)	0.079	-0.24
	18-months	-0.78 (-1.64, 0.09)	0.14 (-0.77, 1.05)	-0.91 (-2.18, 0.35)	0.157	-0.22
Menopausal Symptoms – Vasomotor subscale	Baseline M (SD)	2.57 (2.18)	2.38 (2.10)			
	6-months	0.20 (-0.20, 0.60)	0.55 (0.15, 0.96)	-0.35 (-0.94, 0.23)	0.237	-0.16
	12-months [c]	-0.01 (-0.45, 0.43)	0.46 (0.01, 0.92)	-0.47 (-1.12, 0.17)	0.153	-0.22
	18-months	-0.20 (-0.67, 0.26)	0.33 (-0.16, 0.82)	-0.54 (-1.23, 0.15)	0.127	-0.25
Fear of Cancer Recurrence	Baseline M (SD)	14.43 (9.66)	15.64 (9.73)			
	6-months	-2.00 (-3.61, -0.39)	-1.18 (-2.83, 0.48)	-0.82 (-3.13, 1.49)	0.487	-0.08
	12-months [c]	-2.13 (-3.85, -0.41)	-3.27 (-5.17, -1.37)	1.14 (-1.40, 3.68)	0.379	0.12
	18-months	-2.23 (-4.18, -0.28)	-1.99 (-4.09, 0.10)	-0.24 (-2.97, 2.49)	0.863	-0.02
Body Image – Total score	Baseline M (SD)	2.80 (0.63)	2.74 (0.55)			
	6-months	-0.37 (-0.49, -0.26)	-0.15 (-0.27, -0.03)	-0.22 (-0.39, -0.05)	**0.012**	-0.37
	12-months [c]	-0.46 (-0.59, -0.34)	-0.29 (-0.41, -0.16)	-0.18 (-0.36, -0.00)	**0.048**	-0.30
	18-months	-0.31 (-0.44, -0.17)	-0.24 (-0.39, -0.10)	-0.06 (-0.26, 0.13)	0.521	-0.11

Abbreviations: HDL, high-density lipoprotein; QOL, quality of life.

[a] Extracted from marginal means, with missing data imputed by chained equations (*m*=50 imputations, using predictors of dropout, missing data and auxiliary variables as outlined in eTable 3). Models contain the effects of arm, timepoint (6 / 12 / 18 months), arm*timepoint, and baseline value of the outcome.
[b] Standardized effect: mean intervention effect divided by pooled baseline standard deviation of the outcome.
[c] End-of-intervention contact; primary endpoint.
[d] Higher values are preferable
[e] Modelled as log outcome adjusted for log outcome at baseline, with results back-transformed to change (follow-up minus baseline) in original units using the relevant expression of marginal means.

Table S5. Baseline mean regional body composition measured by dual x-ray absorptiometry in the Living Well after Breast Cancer intervention (n=79) and usual care (n=80) participants

	All (n=143)	Intervention (n=73)	Usual Care (n=70)
		Mean ± SD	
Total body (kg)			
Fat mass	38.21 ± 10.31	38.81 ± 10.41	37.51 ± 10.21
Lean mass	43.21 ± 5.11	42.81 ± 5.01	43.51 ± 5.21
Bone mass	2.41 ± 0.31	2.41 ± 0.31	2.41 ± 0.31
Android region (kg)			
Fat mass	3.21 ± 1.11	3.31 ± 1.21	3.11 ± 1.11
Lean mass	3.11 ± 0.51	3.11 ± 0.41	3.11 ± 0.51
Bone mass	0.01 ± 0.01	0.01 ± 0.01	0.01 ± 0.01
Gynoid region (kg)			
Fat mass	6.71 ± 1.81	6.91 ± 1.81	6.51 ± 1.71
Lean mass	6.61 ± 0.81	6.61 ± 0.81	6.71 ± 0.81
Bone mass	0.21 ± 0.01	0.21 ± 0.01	0.21 ± 0.01
Trunk (kg)			
Fat mass	18.51 ± 5.61	18.81 ± 5.81	18.31 ± 5.41
Lean mass	20.31 ± 2.51	20.11 ± 2.51	20.41 ± 2.51
Bone mass	0.71 ± 0.11	0.71 ± 0.11	0.71 ± 0.11
Legs (kg)			
Fat mass	14.11 ± 4.21	14.51 ± 4.21	13.61 ± 4.21
Lean mass	15.11 ± 2.01	15.01 ± 2.01	15.21 ± 2.11
Bone mass	0.91 ± 0.11	0.91 ± 0.11	0.91 ± 0.11
Arms (kg)			
Fat mass	4.71 ± 1.31	4.61 ± 1.31	4.71 ± 1.41
Lean mass	4.81 ± 0.81	4.71 ± 0.81	5.01 ± 0.81
Bone mass	0.31 ± 0.01	0.31 ± 0.01	0.31 ± 0.01
Total body (% of region)			
Fat mass	45.01 ± 5.31	45.61 ± 5.01	44.41 ± 5.61
Lean mass	52.11 ± 5.01	51.51 ± 4.81	52.71 ± 5.31
Bone mass	2.91 ± 0.41	2.91 ± 0.41	2.91 ± 0.41
Android region (% of region)			
Fat mass	49.11 ± 7.61	49.71 ± 7.21	48.41 ± 7.91
Lean mass	50.21 ± 7.51	49.61 ± 7.11	50.81 ± 7.91
Bone mass	0.71 ± 0.21	0.71 ± 0.21	0.71 ± 0.21
Gynoid region (% of region)			
Fat mass	48.61 ± 5.41	49.61 ± 5.11	47.61 ± 5.51
Lean mass	49.61 ± 5.21	48.71 ± 4.91	50.61 ± 5.41
Bone mass	1.71 ± 0.31	1.71 ± 0.31	1.81 ± 0.31
Trunk (% of region)			
Fat mass	46.11 ± 6.41	46.51 ± 6.11	45.61 ± 6.71
Lean mass	52.11 ± 6.21	51.71 ± 5.91	52.51 ± 6.61
Bone mass	1.81 ± 0.31	1.81 ± 0.31	1.81 ± 0.31
Legs (% of region)			
Fat mass	46.11 ± 5.61	47.01 ± 5.31	45.11 ± 5.81
Lean mass	51.01 ± 5.31	50.11 ± 5.01	52.01 ± 5.41
Bone mass	2.91 ± 0.51	2.91 ± 0.41	3.01 ± 0.51

Arms (% of region)			
Fat mass	47.01 ± 5.21	47.61 ± 4.91	46.51 ± 5.51
Lean mass	49.71 ± 4.81	49.11 ± 4.51	50.21 ± 5.11
Bone mass	3.31 ± 0.61	3.31 ± 0.61	3.31 ± 0.61

Table S6. Within-arm and between-arm changes in regional body composition: Living Well after Breast Cancer trial (evaluable case analyses) [a]

Outcome	Month	Intervention (n=79)			Usual Care (n=80)			Intervention – Usual Care	
		n	Mean change (95% CI)	p	n	Mean change (95% CI)	p	Mean difference (95% CI)	p
Total body (kg)									
Fat mass	6	67	-3.13 (-3.97, -2.30)	<0.001	62	0.13 (-0.73, 1.00)	0.767	-3.26 (-4.47, -2.06)	<0.001
	12	64	-3.27 (-4.26, -2.29)	<0.001	54	0.05 (-0.98, 1.08)	0.924	-3.32 (-4.75, -1.90)	<0.001
	18	63	-2.11 (-3.19, -1.03)	<0.001	54	-0.29 (-1.43, 0.85)	0.617	-1.82 (-3.39, -0.25)	0.023
Lean mass	6	67	-0.96 (-1.28, -0.63)	<0.001	62	-0.24 (-0.57, 0.09)	0.154	-0.71 (-1.18, -0.25)	0.002
	12	64	-1.07 (-1.46, -0.68)	<0.001	54	-0.52 (-0.93, -0.10)	0.015	-0.55 (-1.12, 0.02)	0.059
	18	63	-1.20 (-1.63, -0.77)	<0.001	54	-0.14 (-0.59, 0.32)	0.548	-1.06 (-1.68, -0.43)	<0.001
Bone mass	6	67	-0.03 (-0.04, -0.02)	<0.001	62	-0.02 (-0.03, -0.01)	0.003	-0.01 (-0.03, 0.00)	0.139
	12	64	-0.05 (-0.07, -0.04)	<0.001	54	-0.03 (-0.04, -0.01)	0.002	-0.02 (-0.05, 0.00)	0.028
	18	63	-0.05 (-0.07, -0.03)	<0.001	54	-0.05 (-0.07, -0.03)	<0.001	0.00 (-0.02, 0.03)	0.893
Android region (kg)									
Fat mass	6	67	-0.30 (-0.40, -0.21)	<0.001	62	0.06 (-0.04, 0.16)	0.253	-0.36 (-0.50, -0.22)	<0.001
	12	64	-0.30 (-0.42, -0.19)	<0.001	54	0.07 (-0.05, 0.19)	0.225	-0.38 (-0.54, -0.21)	<0.001
	18	63	-0.20 (-0.33, -0.07)	0.002	54	0.01 (-0.12, 0.15)	0.882	-0.21 (-0.40, -0.02)	0.026
Lean mass	6	67	-0.11 (-0.16, -0.05)	<0.001	62	-0.02 (-0.07, 0.04)	0.528	-0.09 (-0.17, -0.01)	0.022
	12	64	-0.09 (-0.14, -0.03)	0.003	54	-0.08 (-0.14, -0.02)	0.009	-0.01 (-0.09, 0.08)	0.870
	18	63	-0.12 (-0.18, -0.06)	<0.001	54	0.01 (-0.05, 0.07)	0.714	-0.13 (-0.22, -0.05)	0.002
Bone mass	6	67	-0.00 (-0.00, 0.00)	0.903	62	0.00 (0.00, 0.01)	0.001	-0.00 (-0.01, -0.00)	0.017
	12	64	-0.00 (-0.00, 0.00)	0.598	54	0.00 (0.00, 0.00)	0.008	-0.00 (-0.01, -0.00)	0.022
	18	63	-0.00 (-0.00, 0.00)	0.389	54	0.00 (0.00, 0.00)	0.022	-0.00 (-0.01, -0.00)	0.024
Gynoid region (kg)									
Fat mass	6	67	-0.66 (-0.83, -0.50)	<0.001	62	-0.03 (-0.20, 0.14)	0.761	-0.63 (-0.87, -0.40)	<0.001
	12	64	-0.61 (-0.80, -0.42)	<0.001	54	-0.10 (-0.31, 0.10)	0.318	-0.51 (-0.79, -0.23)	<0.001
	18	63	-0.41 (-0.60, -0.21)	<0.001	54	-0.10 (-0.32, 0.11)	0.331	-0.30 (-0.59, -0.01)	0.042
Lean mass	6	67	-0.12 (-0.19, -0.05)	0.002	62	-0.04 (-0.12, 0.04)	0.294	-0.08 (-0.19, 0.03)	0.148
	12	64	-0.17 (-0.25, -0.10)	<0.001	54	-0.11 (-0.19, -0.03)	0.006	-0.06 (-0.17, 0.04)	0.247
	18	63	-0.18 (-0.26, -0.09)	<0.001	54	-0.08 (-0.17, 0.01)	0.073	-0.10 (-0.22, 0.02)	0.117

Bone mass	6	67	-0.00 (-0.01, 0.00)	<0.001	62	-0.00 (-0.01, 0.00)	0.007	-0.00 (-0.00, 0.00)	0.366
	12	64	-0.00 (-0.01, 0.00)	0.064	54	-0.00 (-0.00, 0.00)	0.482	-0.00 (-0.01, 0.00)	0.452
	18	63	-0.00 (-0.01, 0.00)	0.012	54	-0.00 (-0.01, 0.00)	0.045	-0.00 (-0.01, 0.00)	0.801
Trunk (kg)									
Fat mass	6	67	-1.51 (-1.96, -1.06)	<0.001	62	0.15 (-0.32, 0.62)	0.532	-1.66 (-2.31, -1.01)	<0.001
	12	64	-1.62 (-2.15, -1.09)	<0.001	54	0.25 (-0.30, 0.81)	0.371	-1.87 (-2.64, -1.11)	<0.001
	18	63	-0.98 (-1.54, -0.41)	<0.001	54	0.16 (-0.44, 0.75)	0.605	-1.13 (-1.96, -0.31)	0.007
Lean mass	6	67	-0.45 (-0.67, -0.23)	<0.001	62	-0.10 (-0.33, 0.13)	0.402	-0.35 (-0.67, -0.03)	0.034
	12	64	-0.33 (-0.57, -0.09)	0.006	54	-0.33 (-0.58, -0.07)	0.011	0.00 (-0.35, 0.35)	0.991
	18	63	-0.52 (-0.78, -0.26)	<0.001	54	0.05 (-0.23, 0.32)	0.736	-0.57 (-0.94, -0.19)	0.003
Bone mass	6	67	-0.01 (-0.02, 0.00)	0.041	62	0.00 (-0.01, 0.01)	0.947	-0.01 (-0.02, 0.00)	0.143
	12	64	-0.02 (-0.03, -0.01)	<0.001	54	0.01 (-0.01, 0.02)	0.356	-0.03 (-0.04, -0.01)	0.002
	18	63	-0.01 (-0.02, 0.01)	0.434	54	-0.00 (-0.02, 0.01)	0.796	-0.00 (-0.02, 0.02)	0.729
Legs (kg)									
Fat mass	6	67	-1.27 (-1.60, -0.93)	<0.001	62	0.02 (-0.33, 0.36)	0.929	-1.28 (-1.76, -0.80)	<0.001
	12	64	-1.27 (-1.66, -0.87)	<0.001	54	-0.18 (-0.59, 0.24)	0.398	-1.09 (-1.66, -0.52)	<0.001
	18	63	-0.86 (-1.30, -0.42)	<0.001	54	-0.25 (-0.71, 0.22)	0.301	-0.62 (-1.26, 0.02)	0.058
Lean mass	6	67	-0.42 (-0.59, -0.26)	<0.001	62	-0.15 (-0.32, 0.02)	0.086	-0.27 (-0.51, -0.04)	0.022
	12	64	-0.59 (-0.77, -0.41)	<0.001	54	-0.17 (-0.36, 0.02)	0.079	-0.42 (-0.68, -0.16)	0.002
	18	63	-0.53 (-0.73, -0.33)	<0.001	54	-0.09 (-0.30, 0.12)	0.387	-0.44 (-0.73, -0.15)	0.003
Bone mass	6	67	-0.01 (-0.01, 0.00)	<0.001	62	-0.01 (-0.01, 0.00)	<0.001	-0.00 (-0.01, 0.00)	0.627
	12	64	-0.01 (-0.02, -0.01)	<0.001	54	-0.01 (-0.02, -0.01)	<0.001	-0.00 (-0.01, 0.01)	0.871
	18	63	-0.02 (-0.03, -0.02)	<0.001	54	-0.02 (-0.03, -0.01)	<0.001	-0.00 (-0.01, 0.01)	0.532
Arms (kg)									
Fat mass	6	67	-0.36 (-0.48, -0.23)	<0.001	62	-0.06 (-0.19, 0.07)	0.358	-0.30 (-0.48, -0.11)	0.001
	12	64	-0.38 (-0.50, -0.25)	<0.001	54	-0.02 (-0.16, 0.11)	0.723	-0.35 (-0.54, -0.16)	<0.001
	18	63	-0.27 (-0.46, -0.09)	0.003	54	-0.21 (-0.41, -0.02)	0.032	-0.06 (-0.33, 0.20)	0.647
Lean mass	6	67	-0.12 (-0.20, -0.03)	0.006	62	0.01 (-0.08, 0.09)	0.868	-0.13 (-0.25, 0.00)	0.043
	12	64	-0.17 (-0.26, -0.09)	<0.001	54	-0.01 (-0.10, 0.08)	0.823	-0.16 (-0.29, -0.04)	0.008
	18	63	-0.20 (-0.31, -0.09)	<0.001	54	-0.11 (-0.23, 0.00)	0.060	-0.09 (-0.25, 0.07)	0.268
Bone mass	6	67	-0.01 (-0.01, 0.00)	0.002	62	-0.01 (-0.01, 0.00)	0.009	-0.00 (-0.01, 0.01)	0.764
	12	64	-0.01 (-0.01, 0.00)	0.002	54	-0.01 (-0.01, 0.00)	0.016	-0.00 (-0.01, 0.00)	0.706

	18	63	-0.01 (-0.02, 0.00)	<0.001	54	-0.01 (-0.02, -0.01)	<0.001	0.00 (-0.01, 0.01)	0.449
Total body (% of region)									
Fat mass	6	67	-1.93 (-2.55, -1.31)	<0.001	62	0.15 (-0.50, 0.79)	0.658	-2.08 (-2.97, -1.18)	<0.001
	12	64	-1.96 (-2.66, -1.26)	<0.001	54	0.30 (-0.44, 1.03)	0.428	-2.25 (-3.27, -1.24)	<0.001
	18	63	-1.05 (-1.71, -0.38)	0.002	54	-0.08 (-0.78, 0.62)	0.817	-0.96 (-1.93, 0.00)	0.051
Lean mass	6	67	1.81 (1.22, 2.41)	<0.001	62	-0.14 (-0.76, 0.48)	0.663	1.95 (1.09, 2.81)	<0.001
	12	64	1.85 (1.18, 2.52)	<0.001	54	-0.29 (-0.99, 0.41)	0.416	2.14 (1.17, 3.11)	<0.001
	18	63	0.98 (0.34, 1.62)	0.003	54	0.12 (-0.55, 0.79)	0.724	0.86 (-0.07, 1.78)	0.069
Bone mass	6	67	0.12 (0.08, 0.15)	<0.001	62	-0.01 (-0.05, 0.03)	0.652	0.13 (0.07, 0.18)	<0.001
	12	64	0.11 (0.06, 0.15)	<0.001	54	-0.01 (-0.05, 0.04)	0.795	0.12 (0.05, 0.18)	<0.001
	18	63	0.07 (0.02, 0.11)	0.004	54	-0.04 (-0.09, 0.01)	0.092	0.11 (0.04, 0.17)	0.001
Android region (% of region)									
Fat mass	6	67	-2.45 (-3.50, -1.39)	<0.001	62	0.28 (-0.82, 1.37)	0.621	-2.72 (-4.24, -1.20)	<0.001
	12	64	-2.67 (-3.85, -1.49)	<0.001	54	0.84 (-0.40, 2.08)	0.182	-3.52 (-5.23, -1.80)	<0.001
	18	63	-1.36 (-2.49, -0.23)	0.018	54	-0.21 (-1.41, 0.98)	0.724	-1.14 (-2.79, 0.50)	0.173
Lean mass	6	67	2.39 (1.33, 3.44)	<0.001	62	-0.32 (-1.42, 0.77)	0.563	2.71 (1.19, 4.23)	<0.001
	12	64	2.62 (1.44, 3.79)	<0.001	54	-0.88 (-2.12, 0.35)	0.160	3.50 (1.80, 5.21)	<0.001
	18	63	1.32 (0.20, 2.45)	0.021	54	0.18 (-1.00, 1.37)	0.761	1.14 (-0.49, 2.78)	0.172
Bone mass	6	67	0.06 (0.03, 0.09)	<0.001	62	0.05 (0.02, 0.08)	0.001	0.01 (-0.03, 0.05)	0.712
	12	64	0.05 (0.02, 0.08)	0.002	54	0.04 (0.01, 0.07)	0.011	0.01 (-0.04, 0.05)	0.776
	18	63	0.03 (0.00, 0.06)	0.027	54	0.03 (0.00, 0.06)	0.026	-0.00 (-0.04, 0.04)	0.922
Gynoid region (% of region)									
Fat mass	6	67	-2.41 (-3.13, -1.68)	<0.001	62	-0.04 (-0.79, 0.71)	0.916	-2.36 (-3.42, -1.31)	<0.001
	12	64	-2.03 (-2.80, -1.27)	<0.001	54	0.05 (-0.75, 0.85)	0.903	-2.08 (-3.19, -0.97)	<0.001
	18	63	-1.16 (-1.86, -0.47)	<0.001	54	-0.03 (-0.76, 0.70)	0.938	-1.13 (-2.15, -0.12)	0.028
Lean mass	6	67	2.32 (1.61, 3.04)	<0.001	62	0.05 (-0.69, 0.79)	0.891	2.27 (1.24, 3.31)	<0.001
	12	64	1.94 (1.19, 2.69)	<0.001	54	-0.07 (-0.85, 0.71)	0.862	2.01 (0.92, 3.10)	<0.001
	18	63	1.10 (0.43, 1.78)	0.001	54	0.03 (-0.69, 0.75)	0.936	1.07 (0.08, 2.07)	0.034
Bone mass	6	67	0.08 (0.05, 0.10)	<0.001	62	0.00 (-0.03, 0.02)	0.764	0.08 (0.04, 0.12)	<0.001
	12	64	0.09 (0.06, 0.12)	<0.001	54	0.02 (-0.01, 0.06)	0.192	0.07 (0.02, 0.11)	0.006
	18	63	0.05 (0.02, 0.09)	0.002	54	0.00 (-0.03, 0.04)	0.821	0.05 (-0.00, 0.10)	0.053
Trunk (% of region)									

Fat mass	6	67	-2.05 (-2.84, -1.25)	<0.001	62	0.19 (-0.64, 1.01)	0.656	-2.23 (-3.38, -1.09)	<0.001
	12	64	-2.35 (-3.22, -1.48)	<0.001	54	0.56 (-0.35, 1.48)	0.228	-2.91 (-4.18, -1.65)	<0.001
	18	63	-1.12 (-1.98, -0.25)	0.011	54	0.03 (-0.88, 0.94)	0.951	-1.14 (-2.40, 0.11)	0.074
Lean mass	6	67	1.98 (1.18, 2.77)	<0.001	62	-0.19 (-1.01, 0.63)	0.652	2.17 (1.02, 3.31)	<0.001
	12	64	2.31 (1.44, 3.18)	<0.001	54	-0.58 (-1.49, 0.33)	0.208	2.89 (1.64, 4.15)	<0.001
	18	63	1.06 (0.19, 1.92)	0.016	54	-0.02 (-0.93, 0.89)	0.969	1.08 (-0.18, 2.33)	0.093
Bone mass	6	67	0.07 (0.04, 0.10)	<0.001	62	0.00 (-0.03, 0.03)	0.866	0.07 (0.03, 0.11)	<0.001
	12	64	0.04 (0.01, 0.08)	0.018	54	0.02 (-0.02, 0.06)	0.349	0.03 (-0.03, 0.08)	0.344
	18	63	0.06 (0.02, 0.10)	0.002	54	-0.01 (-0.05, 0.03)	0.550	0.07 (0.02, 0.13)	0.010
Legs (% of region)									
Fat mass	6	67	-1.92 (-2.49, -1.34)	<0.001	62	0.20 (-0.40, 0.79)	0.516	-2.11 (-2.95, -1.28)	<0.001
	12	64	-1.68 (-2.35, -1.01)	<0.001	54	0.03 (-0.67, 0.72)	0.943	-1.71 (-2.67, -0.74)	<0.001
	18	63	-0.99 (-1.61, -0.36)	0.002	54	-0.21 (-0.87, 0.45)	0.525	-0.77 (-1.68, 0.14)	0.097
Lean mass	6	67	1.76 (1.22, 2.30)	<0.001	62	-0.19 (-0.76, 0.37)	0.499	1.95 (1.17, 2.74)	<0.001
	12	64	1.52 (0.89, 2.15)	<0.001	54	-0.02 (-0.68, 0.65)	0.959	1.54 (0.62, 2.46)	0.001
	18	63	0.91 (0.32, 1.50)	0.002	54	0.24 (-0.38, 0.87)	0.442	0.67 (-0.19, 1.52)	0.129
Bone mass	6	67	-1.71 (-2.41, -1.01)	<0.001	62	-0.36 (-1.09, 0.36)	0.326	-1.34 (-2.36, -0.33)	0.009
	12	64	-1.53 (-2.28, -0.78)	<0.001	54	-0.01 (-0.80, 0.78)	0.981	-1.52 (-2.61, -0.43)	0.006
	18	63	-0.91 (-1.64, -0.18)	0.015	54	-0.42 (-1.19, 0.36)	0.289	-0.49 (-1.56, 0.58)	0.369
Arms (% of region)									
Fat mass	6	67	0.15 (0.10, 0.20)	<0.001	62	0.00 (-0.05, 0.05)	0.949	0.15 (0.08, 0.22)	<0.001
	12	64	0.15 (0.10, 0.21)	<0.001	54	-0.01 (-0.06, 0.05)	0.861	0.16 (0.08, 0.24)	<0.001
	18	63	0.07 (0.01, 0.13)	0.014	54	-0.03 (-0.09, 0.03)	0.350	0.10 (0.02, 0.19)	0.018
Lean mass	6	67	1.60 (0.91, 2.29)	<0.001	62	0.40 (-0.32, 1.11)	0.277	1.20 (0.20, 2.20)	0.018
	12	64	1.38 (0.64, 2.12)	<0.001	54	0.04 (-0.74, 0.82)	0.923	1.34 (0.27, 2.41)	0.014
	18	63	0.83 (0.11, 1.55)	0.023	54	0.47 (-0.29, 1.23)	0.227	0.36 (-0.69, 1.41)	0.498
Bone mass	6	67	0.11 (0.05, 0.16)	<0.001	62	-0.02 (-0.08, 0.03)	0.426	0.13 (0.05, 0.21)	0.002
	12	64	0.14 (0.08, 0.21)	<0.001	54	-0.02 (-0.09, 0.05)	0.564	0.16 (0.07, 0.26)	<0.001
	18	63	0.07 (-0.00, 0.14)	0.065	54	-0.04 (-0.12, 0.03)	0.258	0.11 (0.01, 0.21)	0.037

[a] Mean changes and intervention effects estimated from mixed models with fixed terms for randomisation arm (intervention / control), timepoint (6/12/18 months), randomisation arm#timepoint, and baseline value of the outcome, and random terms for timepoint (6 / 12 /18 months) and participant. Table reports changes and differences in changes from marginal means estimated at means of baseline values.

Table S7. Within-arm and between-arm changes in regional body composition: Living Well after Breast Cancer trial (multiple imputation analyses)[a]

Outcome (Baseline M ± SE)	Month	Intervention (n=79) [b]		Usual Care (n=80) [b]		Intervention – Usual Care [b]	
		Mean change (95% CI)	p	Mean change (95% CI)	p	Mean difference (95% CI)	p
Total body (kg)							
Fat mass	6	-3.07 (-4.06, -2.09)	<0.001	0.22 (-0.83, 1.26)	0.680	-3.29 (-4.73, -1.85)	<0.001
(38.59 ± 0.83)	12	-3.23 (-4.37, -2.09)	<0.001	0.24 (-1.01, 1.49)	0.708	-3.47 (-5.12, -1.81)	<0.001
	18	-2.06 (-3.29, -0.83)	0.001	-0.63 (-1.92, 0.65)	0.335	-1.42 (-3.19, 0.34)	0.114
Lean mass	6	-1.03 (-1.40, -0.66)	<0.001	-0.27 (-0.67, 0.13)	0.183	-0.75 (-1.28, -0.23)	0.005
(43.35 ± 0.42)	12	-1.10 (-1.55, -0.66)	<0.001	-0.49 (-1.04, 0.05)	0.073	-0.61 (-1.27, 0.05)	0.071
	18	-1.24 (-1.73, -0.75)	<0.001	-0.17 (-0.72, 0.38)	0.550	-1.07 (-1.78, -0.37)	0.003
Bone mass	6	-0.03 (-0.05, -0.02)	<0.001	-0.02 (-0.03, -0.00)	0.043	-0.02 (-0.04, 0.00)	0.097
(2.40 ± 0.03)	12	-0.05 (-0.07, -0.03)	<0.001	-0.03 (-0.05, -0.01)	0.004	-0.02 (-0.05, 0.01)	0.151
	18	-0.05 (-0.07, -0.03)	<0.001	-0.06 (-0.08, -0.03)	<0.001	0.01 (-0.02, 0.04)	0.662
Android region (kg)							
Fat mass	6	-0.30 (-0.42, -0.18)	<0.001	0.08 (-0.05, 0.21)	0.230	-0.37 (-0.55, -0.20)	<0.001
(3.23 ± 0.09)	12	-0.29 (-0.43, -0.16)	<0.001	0.08 (-0.07, 0.23)	0.295	-0.37 (-0.57, -0.17)	<0.001
	18	-0.20 (-0.35, -0.04)	0.012	-0.03 (-0.19, 0.13)	0.701	-0.17 (-0.39, 0.05)	0.137
Lean mass	6	-0.11 (-0.18, -0.05)	<0.001	0.00 (-0.06, 0.07)	0.963	-0.12 (-0.20, -0.03)	0.009
(3.12 ± 0.04)	12	-0.08 (-0.15, -0.02)	0.016	-0.06 (-0.14, 0.02)	0.136	-0.02 (-0.12, 0.08)	0.704
	18	-0.12 (-0.19, -0.05)	<0.001	0.03 (-0.05, 0.11)	0.407	-0.15 (-0.25, -0.05)	0.003
Bone mass	6	0.00 (-0.00, 0.00)	0.972	0.00 (0.00, 0.01)	0.006	0.00 (-0.01, 0.00)	0.037
(0.05 ± 0.00)	12	-0.00 (-0.00, 0.00)	0.797	0.00 (-0.00, 0.01)	0.072	0.00 (-0.01, 0.00)	0.103
	18	-0.00 (-0.00, 0.00)	0.569	0.00 (-0.00, 0.00)	0.271	0.00 (-0.01, 0.00)	0.202
Gynoid region (kg)							
Fat mass	6	-0.62 (-0.82, -0.42)	<0.001	0.02 (-0.20, 0.24)	0.867	-0.64 (-0.94, -0.34)	<0.001
(6.71 ± 0.15)	12	-0.58 (-0.81, -0.35)	<0.001	0.00 (-0.26, 0.26)	0.998	-0.58 (-0.93, -0.24)	<0.001

	18	-0.39 (-0.62, -0.16)	0.001	-0.18 (-0.43, 0.07)	0.161	-0.21 (-0.55, 0.13)	0.224
Lean mass	6	-0.13 (-0.22, -0.04)	0.003	-0.06 (-0.15, 0.03)	0.186	-0.07 (-0.19, 0.05)	0.272
(6.66 ± 0.07)	12	-0.18 (-0.26, -0.09)	<0.001	-0.13 (-0.23, -0.03)	0.010	-0.05 (-0.17, 0.08)	0.461
	18	-0.18 (-0.28, -0.08)	<0.001	-0.12 (-0.23, -0.01)	0.036	-0.06 (-0.20, 0.08)	0.412
Bone mass	6	-0.01 (-0.01, 0.00)	<0.001	-0.00 (-0.00, 0.00)	0.448	-0.00 (-0.01, 0.00)	0.071
(0.23 ± 0.00)	12	-0.00 (-0.01, 0.00)	0.088	0.00 (-0.00, 0.01)	0.537	-0.01 (-0.01, 0.00)	0.111
	18	-0.00 (-0.01, 0.00)	0.015	-0.00 (-0.01, 0.00)	0.079	0.00 (-0.01, 0.01)	0.777
Trunk (kg)							
Fat mass	6	-1.44 (-2.00, -0.89)	<0.001	0.19 (-0.41, 0.78)	0.539	-1.63 (-2.44, -0.82)	<0.001
(18.74 ± 0.45)	12	-1.54 (-2.16, -0.92)	<0.001	0.29 (-0.41, 0.98)	0.420	-1.82 (-2.73, -0.92)	<0.001
	18	-0.97 (-1.65, -0.29)	0.005	-0.12 (-0.83, 0.59)	0.745	-0.85 (-1.83, 0.12)	0.087
Lean mass	6	-0.47 (-0.73, -0.21)	<0.001	-0.09 (-0.37, 0.20)	0.552	-0.39 (-0.75, -0.02)	0.041
(20.35 ± 0.21)	12	-0.34 (-0.64, -0.05)	0.021	-0.37 (-0.73, 0.00)	0.048	0.02 (-0.41, 0.46)	0.920
	18	-0.50 (-0.82, -0.18)	0.002	0.02 (-0.35, 0.38)	0.930	-0.52 (-0.97, -0.06)	0.027
Bone mass	6	-0.01 (-0.02, 0.00)	0.077	0.00 (-0.01, 0.01)	0.923	-0.01 (-0.03, 0.01)	0.200
(0.71 ± 0.01)	12	-0.02 (-0.03, -0.01)	0.001	0.00 (-0.02, 0.02)	0.911	-0.02 (-0.04, -0.00)	0.032
	18	-0.01 (-0.02, 0.01)	0.399	-0.01 (-0.03, 0.01)	0.291	0.00 (-0.02, 0.03)	0.781
Legs (kg)							
Fat mass	6	-1.20 (-1.61, -0.80)	<0.001	-0.07 (-0.51, 0.37)	0.763	-1.14 (-1.74, -0.53)	<0.001
(14.26 ± 0.34)	12	-1.24 (-1.70, -0.78)	<0.001	-0.14 (-0.66, 0.38)	0.602	-1.10 (-1.79, -0.42)	0.002
	18	-0.78 (-1.29, -0.27)	0.003	-0.37 (-0.92, 0.17)	0.177	-0.41 (-1.15, 0.34)	0.288
Lean mass	6	-0.46 (-0.65, -0.27)	<0.001	-0.19 (-0.39, 0.01)	0.063	-0.27 (-0.53, 0.00)	0.052
(15.21 ± 0.17)	12	-0.62 (-0.82, -0.41)	<0.001	-0.14 (-0.38, 0.10)	0.243	-0.47 (-0.77, -0.18)	0.002
	18	-0.56 (-0.79, -0.33)	<0.001	-0.12 (-0.37, 0.14)	0.381	-0.45 (-0.78, -0.12)	0.008
Bone mass	6	-0.01 (-0.01, -0.00)	<0.001	-0.01 (-0.01, -0.00)	0.021	-0.00 (-0.01, 0.00)	0.507
(0.86 ± 0.01)	12	-0.01 (-0.02, -0.01)	<0.001	-0.01 (-0.02, -0.01)	<0.001	-0.00 (-0.01, 0.01)	0.939
	18	-0.02 (-0.03, -0.02)	<0.001	-0.02 (-0.03, -0.01)	<0.001	-0.00 (-0.01, 0.01)	0.563
Arms (kg)							
Fat mass	6	-0.34 (-0.49, -0.20)	<0.001	-0.02 (-0.17, 0.14)	0.843	-0.33 (-0.54, -0.12)	0.002
(4.71 ± 0.11)	12	-0.37 (-0.52, -0.21)	<0.001	0.00 (-0.17, 0.18)	0.980	-0.37 (-0.59, -0.15)	0.001
	18	-0.23 (-0.45, -0.01)	0.040	-0.18 (-0.41, 0.05)	0.125	-0.05 (-0.37, 0.26)	0.749

Lean mass	6	-0.15 (-0.24, -0.05)	0.003	0.05 (-0.05, 0.15)	0.357	-0.19 (-0.33, -0.06)	0.005
(4.85 ± 0.07)	12	-0.18 (-0.28, -0.09)	<0.001	0.04 (-0.06, 0.15)	0.425	-0.23 (-0.37, -0.09)	0.001
	18	-0.21 (-0.34, -0.08)	0.002	-0.05 (-0.20, 0.09)	0.472	-0.15 (-0.35, 0.04)	0.117
Bone mass	6	-0.01 (-0.01, 0.00)	0.003	-0.01 (-0.01, 0.00)	0.063	-0.00 (-0.01, 0.00)	0.521
(0.32 ± 0.00)	12	-0.01 (-0.01, 0.00)	0.003	-0.01 (-0.01, -0.00)	0.048	-0.00 (-0.01, 0.01)	0.734
	18	-0.01 (-0.02, 0.00)	0.005	-0.01 (-0.02, -0.00)	0.004	0.00 (-0.01, 0.01)	0.757
Total body (% of region)							
Fat mass	6	-1.81 (-2.48, -1.13)	<0.001	0.21 (-0.52, 0.94)	0.571	-2.02 (-3.02, -1.02)	<0.001
(45.14 ± 0.44)	12	-1.87 (-2.64, -1.09)	<0.001	0.39 (-0.52, 1.30)	0.397	-2.26 (-3.42, -1.10)	<0.001
	18	-0.97 (-1.75, -0.19)	0.015	-0.35 (-1.21, 0.51)	0.428	-0.62 (-1.77, 0.52)	0.286
Lean mass	6	1.69 (1.05, 2.34)	<0.001	-0.20 (-0.91, 0.50)	0.570	1.90 (0.93, 2.86)	<0.001
(51.97 ± 0.42)	12	1.76 (1.02, 2.50)	<0.001	-0.37 (-1.25, 0.51)	0.407	2.13 (1.02, 3.25)	<0.001
	8	0.90 (0.16, 1.65)	0.018	0.38 (-0.45, 1.21)	0.371	0.53 (-0.58, 1.63)	0.349
Bone mass	6	0.11 (0.07, 0.15)	<0.001	-0.01 (-0.05, 0.04)	0.783	0.12 (0.06, 0.18)	<0.001
(2.89 ± 0.03)	12	0.10 (0.05, 0.15)	<0.001	-0.02 (-0.07, 0.03)	0.487	0.12 (0.05, 0.19)	<0.001
	18	0.06 (0.01, 0.11)	0.012	-0.03 (-0.08, 0.02)	0.264	0.09 (0.02, 0.17)	0.010
Android region (% of region)							
Fat mass	6	-2.26 (-3.47, -1.06)	<0.001	0.15 (-1.17, 1.47)	0.824	-2.41 (-4.21, -0.62)	0.008
(49.26 ± 0.63)	12	-2.54 (-4.09, -1.00)	0.001	0.54 (-1.52, 2.59)	0.608	-3.08 (-5.57, -0.59)	0.016
	18	-1.34 (-2.78, 0.10)	0.068	-1.01 (-2.71, 0.69)	0.242	-0.33 (-2.50, 1.84)	0.766
Lean mass	6	2.20 (1.00, 3.41)	<0.001	-0.19 (-1.52, 1.13)	0.777	2.39 (0.60, 4.18)	0.009
(50.02 ± 0.62)	12	2.49 (0.96, 4.02)	0.001	-0.57 (-2.61, 1.46)	0.580	3.06 (0.59, 5.54)	0.015
	18	1.30 (-0.13, 2.74)	0.076	0.99 (-0.71, 2.69)	0.251	0.31 (-1.86, 2.48)	0.779
Bone mass	6	0.05 (0.02, 0.09)	0.002	0.05 (0.01, 0.09)	0.010	0.00 (-0.05, 0.05)	0.867
(0.72 ± 0.02)	12	0.05 (0.00, 0.09)	0.029	0.05 (-0.00, 0.10)	0.073	-0.00 (-0.07, 0.06)	0.968
	18	0.03 (-0.01, 0.07)	0.096	0.03 (-0.02, 0.07)	0.216	0.00 (-0.05, 0.06)	0.928
Gynoid region (% of region)							
Fat mass	6	-2.17 (-2.98, -1.36)	<0.001	0.15 (-0.73, 1.03)	0.743	-2.32 (-3.52, -1.11)	<0.001
(48.66 ± 0.45)	12	-1.86 (-2.74, -0.97)	<0.001	0.39 (-0.65, 1.42)	0.463	-2.24 (-3.56, -0.92)	<0.001
	18	-1.07 (-1.93, -0.20)	0.016	-0.33 (-1.26, 0.61)	0.496	-0.74 (-2.01, 0.53)	0.254
Lean mass	6	2.09 (1.29, 2.89)	<0.001	-0.15 (-1.01, 0.72)	0.740	2.24 (1.05, 3.42)	<0.001

(49.62 ± 0.44)	12	1.76 (0.90, 2.63)	<0.001	-0.41 (-1.44, 0.61)	0.428	2.18 (0.88, 3.48)	0.001
	18	1.01 (0.15, 1.86)	0.021	0.32 (-0.61, 1.24)	0.503	0.69 (-0.56, 1.94)	0.278
Bone mass	6	0.07 (0.03, 0.10)	<0.001	0.01 (-0.03, 0.05)	0.546	0.05 (0.00, 0.11)	0.040
(1.72 ± 0.02)	12	0.08 (0.04, 0.12)	<0.001	0.04 (-0.00, 0.08)	0.074	0.04 (-0.02, 0.10)	0.177
	18	0.05 (0.00, 0.09)	0.035	0.02 (-0.02, 0.07)	0.352	0.02 (-0.04, 0.08)	0.457
Trunk (% of region)							
Fat mass	6	-1.89 (-2.79, -1.00)	<0.001	0.17 (-0.81, 1.14)	0.735	-2.06 (-3.39, -0.73)	0.002
(46.25 ± 0.53)	12	-2.17 (-3.16, -1.19)	<0.001	0.63 (-0.56, 1.81)	0.297	-2.80 (-4.28, -1.32)	<0.001
	18	-1.12 (-2.17, -0.07)	0.036	-0.37 (-1.53, 0.78)	0.524	-0.75 (-2.28, 0.79)	0.341
Lean mass	6	1.83 (0.93, 2.72)	<0.001	-0.17 (-1.14, 0.80)	0.732	2.00 (0.67, 3.32)	0.003
(51.94 ± 0.51)	12	2.14 (1.16, 3.11)	<0.001	-0.64 (-1.83, 0.54)	0.288	2.78 (1.30, 4.25)	<0.001
	18	1.07 (0.02, 2.11)	0.046	0.39 (-0.77, 1.55)	0.510	0.68 (-0.86, 2.22)	0.388
Bone mass	6	0.07 (0.03, 0.10)	<0.001	0.00 (-0.03, 0.04)	0.859	0.06 (0.02, 0.11)	0.005
(1.81 ± 0.02)	12	0.04 (-0.00, 0.08)	0.062	0.01 (-0.03, 0.06)	0.575	0.02 (-0.03, 0.08)	0.410
	18	0.05 (0.01, 0.10)	0.015	-0.01 (-0.07, 0.04)	0.569	0.07 (0.00, 0.13)	0.036
Legs (% of region)							
Fat mass	6	-1.73 (-2.38, -1.07)	<0.001	0.12 (-0.60, 0.84)	0.748	-1.85 (-2.83, -0.86)	<0.001
(46.27 ± 0.47)	12	-1.57 (-2.35, -0.78)	<0.001	-0.01 (-0.93, 0.91)	0.983	-1.56 (-2.75, -0.36)	0.011
	18	-0.78 (-1.57, 0.00)	0.051	-0.47 (-1.35, 0.40)	0.289	-0.31 (-1.49, 0.87)	0.608
Lean mass	6	1.58 (0.96, 2.19)	<0.001	-0.13 (-0.81, 0.55)	0.706	1.71 (0.78, 2.64)	<0.001
(50.83 ± 0.44)	12	1.41 (0.67, 2.16)	<0.001	0.03 (-0.86, 0.91)	0.954	1.39 (0.25, 2.52)	0.017
	18	0.71 (-0.04, 1.45)	0.063	0.48 (-0.35, 1.32)	0.253	0.22 (-0.89, 1.34)	0.697
Bone mass	6	0.15 (0.09, 0.21)	<0.001	0.02 (-0.04, 0.08)	0.574	0.13 (0.04, 0.21)	0.003
(2.91 ± 0.04)	12	0.15 (0.09, 0.21)	<0.001	-0.01 (-0.08, 0.05)	0.742	0.16 (0.07, 0.25)	<0.001
	18	0.07 (0.00, 0.14)	0.038	-0.01 (-0.08, 0.07)	0.861	0.08 (-0.02, 0.18)	0.122
Arms (% of region)							
Fat mass	6	-1.43 (-2.23, -0.64)	<0.001	-0.34 (-1.24, 0.56)	0.462	-1.10 (-2.26, 0.07)	0.066
(47.10 ± 0.47)	12	-1.34 (-2.25, -0.43)	0.004	-0.19 (-1.36, 0.98)	0.748	-1.15 (-2.53, 0.23)	0.101
	18	-0.60 (-1.61, 0.42)	0.249	-0.75 (-1.97, 0.48)	0.232	0.15 (-1.39, 1.69)	0.848
Lean mass	6	1.32 (0.54, 2.09)	<0.001	0.42 (-0.47, 1.30)	0.353	0.90 (-0.24, 2.04)	0.123
(49.57 ± 0.44)	12	1.20 (0.31, 2.08)	0.008	0.27 (-0.88, 1.43)	0.642	0.92 (-0.44, 2.28)	0.183

	18	0.52 (-0.46, 1.51)	0.297	0.81 (-0.38, 2.01)	0.181	-0.29 (-1.79, 1.21)	0.704
Bone mass	6	0.10 (0.03, 0.18)	0.006	-0.06 (-0.15, 0.02)	0.147	0.17 (0.05, 0.28)	0.004
(3.34 ± 0.05)	12	0.13 (0.06, 0.21)	<0.001	-0.07 (-0.16, 0.02)	0.145	0.20 (0.08, 0.32)	<0.001
	18	0.06 (-0.03, 0.14)	0.211	-0.05 (-0.16, 0.06)	0.336	0.11 (-0.03, 0.25)	0.120

[a] Six multiple imputation datasets (one per body region) were created using the multivariate normal method (STATA mi impute mvn) with m=70 imputations (adequate to cover maximum fraction of missing information). Each imputation model contained all analytic variables plus baseline predictors of missing DXA data (p<0.2), and auxiliary variables – specifically variables with p<0.2 association with changes in body weight or the composition of any of the six body regions (p<0.2). Specifically each imputation model contained the analytic variables plus: Depression (T score); Quality of Life Physical Health Component (T score); Mastectomy (yes / no); Positive Lymph Nodes (yes / no); Stage 2 Breast Cancer (yes/no); Stage 3 Breast Cancer (yes/no); Endocrine Treatment (none / aromatase inhibitor / other); Treatment (surgery only / surgery + chemotherapy / surgery + radiotherapy / surgery + radiotherapy + chemotherapy); Breast cancer subtype (HR+ and HER2- / HR+ and HER2- / HR+ and HER2+ / HR- and HER2+ / unknown); Ethnicity (Caucasian / other); Depression and/or anxiety (yes / no); Lymphoedema (yes / no); Time Since Diagnosis (months); Education (≤ high school / technical or diploma / ≥ university); Country of Birth (Australia / other); Menopausal status at diagnosis (Premenopausal / Perimenopausal / Postmenopausal); Charlson Comorbidity Index (0 / 1 / 2 / 3 / ≥4); Weekly gross household income (AUD: <$82;056 / ≥$82;056 / unknown); body mass index (kg/m^2); Diabetes (yes/no); Children <18 at home (yes / no); Prior use of weight loss aids (yes/no).

[b] Mean changes and intervention effects were estimated from mixed models with fixed terms for randomisation arm (intervention / control), timepoint (6/12/18 months), randomisation arm #timepoint, and baseline value of the outcome, and random terms for timepoint (6 / 12 /18 months) and participant. Marginal means were evaluated at pooled baseline mean (reported in table).

Table S8. Changes within each arm and intervention effects on medication-sensitive outcomes, adjusted for baseline and concurrent use of relevant medications in the Living Well after Breast Cancer Trial

**Outcome**	**Timepoint**	**Intervention**		**Usual Care**		**Intervention effect (Intervention – Usual care)**		
		**n**	**Change (95% CI)** [a]	**n**	**Change (95% CI)** [a]	**Intervention Effect** [a]	***p***	**d** [b]
Metabolic Syndrome risk score *[blood pressure and lipid lowering medication; endocrine treatment]*	Baseline M (SD)	78	0.65 (0.60)	77	0.63 (0.59)			
	6 months	69	-0.20 (-0.28, -0.12)	65	0.03 (-0.05, 0.12)	-0.23 (-0.35, -0.11)	**<0.001**	-0.39
	12 months [c]	67	-0.18 (-0.27, -0.09)	55	0.00 (-0.10, 0.10)	-0.18 (-0.32, -0.05)	**0.008**	-0.31
	18 months	64	-0.15 (-0.24, -0.06)	56	0.01 (-0.09, 0.10)	-0.16 (-0.29, -0.03)	**0.019**	-0.26
Triglycerides (mmol/L) [d] *[lipid lowering medication; endocrine treatment]*	Baseline M (SD)	78	1.42 (0.71)	78	1.52 (0.88)			
	6 months	71	-0.04 (-0.12, 0.04)	67	0.08 (-0.02, 0.17)	-0.12 (-0.24, 0.01)	0.069	-0.15
	12 months [c]	67	-0.08 (-0.18, 0.02)	56	0.03 (-0.08, 0.15)	-0.11 (-0.27, 0.04)	0.147	-0.14
	18 months	64	-0.11 (-0.20, -0.02)	58	-0.01 (-0.10, 0.09)	-0.10 (-0.24, 0.03)	0.125	-0.13
HDL-cholesterol (mmol/L) [e] *[lipid lowering medication; endocrine treatment]*	Baseline M (SD)	78	1.45 (0.30)	78	1.42 (0.35)			
	6 months	71	0.03 (-0.02, 0.07)	67	-0.02 (-0.06, 0.03)	0.04 (-0.02, 0.10)	0.176	0.13
	12 months [c]	67	0.04 (-0.00, 0.09)	56	-0.00 (-0.05, 0.05)	0.05 (-0.02, 0.12)	0.171	0.15
	18 months	64	0.06 (0.02, 0.11)	58	0.01 (-0.04, 0.06)	0.05 (-0.01, 0.12)	0.114	0.16
Systolic blood pressure (mmHg) *[blood pressure lowering medication]*	Baseline M (SD)	79	125.27 (12.18)	79	123.39 (11.33)			
	6 months	71	-1.71 (-4.25, 0.82)	67	3.42 (0.81, 6.04)	-5.14 (-8.79, -1.49)	**0.006**	-0.44
	12 months [c]	70	1.03 (-1.75, 3.80)	59	2.18 (-0.81, 5.17)	-1.15 (-5.24, 2.93)	0.579	-0.10
	18 months	67	2.94 (-0.53, 6.40)	59	5.51 (1.85, 9.17)	-2.57 (-7.62, 2.48)	0.318	-0.22
Diastolic blood pressure (mmHg) *[blood pressure lowering medication]*	Baseline M (SD)	79	78.66 (9.38)	79	77.95 (7.29)			
	6 months	71	-0.37 (-2.06, 1.32)	67	2.44 (0.70, 4.18)	-2.82 (-5.24, -0.39)	**0.023**	-0.34
	12 months [c]	70	0.59 (-1.09, 2.27)	59	1.52 (-0.28, 3.33)	-0.94 (-3.41, 1.53)	0.458	-0.11
	18 months	67	1.09 (-0.80, 2.97)	59	3.40 (1.41, 5.40)	-2.31 (-5.07, 0.44)	0.099	-0.28

Table S9. Changes within each arm and intervention effects on medication-sensitive outcomes, adjusted for baseline and concurrent use of relevant medications in the Living Well after Breast Cancer Trial (continued)

Outcome	Timepoint	Intervention		Usual Care		Intervention effect (Intervention – Usual care)		
		**n**	**Change (95% CI)** [a]	**n**	**Change (95% CI)** [a]	**Intervention Effect** [a]	***p***	**d** [b]
Musculoskeletal Pain *[endocrine treatment]*	Baseline M (SD)	63	1.55 (1.06)	59	1.63 (1.05)			
	6 months	56	-0.20 (-0.42, 0.02)	50	0.40 (0.16, 0.63)	-0.60 (-0.92, -0.28)	**<0.001**	-0.57
	12 months [c]	52	-0.18 (-0.41, 0.05)	45	0.31 (0.06, 0.56)	-0.49 (-0.83, -0.15)	**0.005**	-0.47
	18 months	52	-0.04 (-0.29, 0.20)	43	0.26 (-0.01, 0.53)	-0.30 (-0.67, 0.06)	0.101	-0.29
Menopausal Symptoms – Psychological subscale *[endocrine treatment]*	Baseline M (SD)	75	10.01 (6.20)	76	9.63 (5.69)			
	6 months	66	-1.44 (-2.55, -0.33)	65	-0.11 (-1.23, 1.01)	-1.33 (-2.91, 0.25)	0.099	-0.22
	12 months [c]	62	-1.88 (-3.16, -0.60)	57	-0.77 (-2.10, 0.56)	-1.11 (-2.96, 0.75)	0.2417	-0.19
	18 months	60	-1.24 (-2.54, 0.05)	55	-0.24 (-1.57, 1.09)	-1.00 (-2.86, 0.86)	0.293	-0.17
Menopausal Symptoms – Somatic subscale *[endocrine treatment]*	Baseline M (SD)	76	5.53 (4.31)	76	5.08 (4.00)			
	6 months	67	-0.68 (-1.32, -0.05)	65	0.59 (-0.05, 1.24)	-1.28 (-2.19, -0.37)	**0.006**	-0.31
	12 months [c]	62	-0.77 (-1.53, -0.02)	57	-0.13 (-0.91, 0.66)	-0.65 (-1.74, 0.45)	0.246	-0.16
	18 months	62	-0.63 (-1.45, 0.19)	55	0.22 (-0.63, 1.08)	-0.85 (-2.04, 0.34)	0.161	-0.20
Menopausal Symptoms – Vasomotor subscale *[endocrine treatment]*	Baseline M (SD)	76	2.57 (2.16)	76	2.37 (2.09)			
	6 months	67	0.20 (-0.16, 0.55)	65	0.53 (0.17, 0.89)	-0.33 (-0.84, 0.17)	0.197	-0.16
	12 months [c]	63	0.05 (-0.35, 0.45)	57	0.39 (-0.03, 0.81)	-0.34 (-0.92, 0.24)	0.255	-0.16
	18 months	62	-0.20 (-0.61, 0.22)	55	0.32 (-0.12, 0.76)	-0.52 (-1.12, 0.09)	0.096	-0.24

Abbreviations: HDL, high density lipoprotein

[a] mean change or mean difference in change (95 CI%), extracted from marginal means. Models include effects of arm, timepoint (6 / 12 /18), arm*timepoint, baseline value of the outcome, use of medications noted in the table at baseline and concurrently: lipid-lowering medication (yes/no); blood pressure lowering medication (yes/no); and, endocrine treatment (none / aromatase inhibitors / other). Other endocrine treatments were mostly selective estrogen receptor modulators and one case of gonadotropin releasing hormone agonist.

[b] Standardized effect: mean intervention effect divided by pooled baseline standard deviation of the outcome.

[c] End-of-intervention contact; primary endpoint.
[d] Modelled as log outcome adjusted for log outcome at baseline, with results back-transformed to change (follow-up minus baseline) in original units using the relevant expression of marginal means.
[e] Higher values are preferable.

Supplementary Material References

1. Alberti KG, Eckel RH, Grundy SM, et al. Harmonizing the metabolic syndrome: a joint interim statement of the International Diabetes Federation Task Force on Epidemiology and Prevention; National Heart, Lung, and Blood Institute; American Heart Association; World Heart Federation; International Atherosclerosis Society; and International Association for the Study of Obesity. *Circulation*. Oct 20 2009;120(16):1640-5. doi:10.1161/circulationaha.109.192644

2. Wijndaele K, Healy GN, Dunstan DW, et al. Increased cardiometabolic risk is associated with increased TV viewing time. *Med Sci Sports Exerc*. Aug 2010;42(8):1511-8. doi:10.1249/MSS.0b013e3181d322ac

3. Ekelund U, Griffin SJ, Wareham NJ. Physical activity and metabolic risk in individuals with a family history of type 2 diabetes. *Diabetes care*. Feb 2007;30(2):337-42. doi:10.2337/dc06-1883

4. Healy GN, Winkler EAH, Owen N, Anuradha S, Dunstan DW. Replacing sitting time with standing or stepping: associations with cardio-metabolic risk biomarkers. *European Heart Journal*. 2015;36(39):2643-2649. doi:10.1093/eurheartj/ehv308
